# Real-World Experience of Coronary Bifurcation Stenting in a Single Centre in Western India

**DOI:** 10.7759/cureus.107216

**Published:** 2026-04-17

**Authors:** Avinash D Arke, Akshay Kawadkar, Shishir Kumar Roul, Tirichala Rajesh, Akkarapaka Rinita Ajey

**Affiliations:** 1 Cardiology, Jagjivanram Hospital, Mumbai, IND

**Keywords:** bifurcation stenting, coronary artery disease, coronary bifurcation lesions, culotte technique, dk crush technique, main branch, provisional stenting technique, side branch, two stent strategy

## Abstract

Background: Coronary bifurcation lesions (CBLs) are recognised as an intricate form of coronary artery disease. The anatomic complexity and variable flow dynamics at branch points pose unique challenges, often contributing to suboptimal outcomes if not managed appropriately. Introduction of drug-eluting stents (DESs) in percutaneous coronary interventions (PCIs) practice had been a major breakthrough. However, there are some concerns in bifurcation coronary stenting like choice of bifurcation stenting technique and issue related to side branch (SB) occlusion. This study aimed to present the single-centre real-world experience with bifurcation stenting, highlighting procedural strategies, outcomes and learning points from routine clinical practice.

Methods: This descriptive observational study was carried out at a cardiac centre located in western India.

Results: Among the 70 patients who underwent PCI for CBL, 72.8% were male, mean age being 65.6 ± 2.8 years. Most frequently encountered CBL was in left anterior descending (LAD)/diagonal (n=24, 34.3%). Out of 70 patients, most prevalent CBL subtypes were Medina 1,1,1 (n = 26, 37.1%) and Medina 1,1,0 (n=20, 28.6%), reflecting the predominance of complex anatomical involvement. The most common coronary bifurcation technique utilised was provisional stenting (PS) (n=46, 65.7%), followed by Double-Kissing (DK) crush technique (n=12, 17.1%). Major adverse cardiac events (MACE) at one month, six months and one year occurred in 4.3% (95% confidence interval (CI): 1.5-11.9), 7.1% (95% CI: 3.1-15.8) and 7.1% (95% CI: 3.1-15.8) patients, respectively; there was no statistical significance.

Conclusion: Coronary bifurcation stenting (COBIS), when guided by lesion anatomy, sound strategy selection and meticulous technique, yielded high procedural success and favourable clinical outcomes.

## Introduction

Percutaneous coronary interventions (PCIs) with stenting with drug-eluting stent (DES) is the preferred strategy to treat a significantly stenotic coronary artery disease. Bifurcation lesions are complex form of coronary artery disease, encountered in up to 15-20% of all PCI [1]. The treatment of coronary bifurcation lesion (CBL) poses a difficulty because of the complexity of the interventions. PCIs for CBLs were found to have poor outcomes as compared to the PCIs to other lesions [2,3]. The anatomic complexity and variable flow dynamics at branch points pose unique challenges [4]. The concerns in bifurcation coronary stenting are provisional stenting (PS) verses afferent two stent strategies: choice of bifurcation stenting technique and issue related to side branch (SB) occlusion. There are various bifurcation stenting techniques for managing CBL, and their clinical outcomes were evaluated in multiple randomised controlled trials (RCTs). Proximal optimisation technique (POT) and final kissing balloon (FKB) have shown better outcomes in stenting CBLs [5,6]. The latest guidelines on myocardial revascularisation recommend PS as the first-choice approach for PCI of CBLs [7,8].

We aimed to present the single-centre real-world experience with bifurcation stenting, highlighting procedural strategies, outcomes, and learning points from routine clinical practice. The primary endpoint measured was occurrence of major adverse cardiac events (MACE), defined as myocardial infarction (MI), target lesion revascularization (TLR) or stent thrombosis. All-cause mortality during the follow-up period was assessed as a secondary endpoint.

## Materials and methods

Study design

This prospective descriptive observational study was carried out in a tertiary care referral hospital in Mumbai, India, from June 2023 to May 2025, which included the phases of patient recruitment, coronary interventions using bifurcation techniques, short-term monitoring as well as long-term follow-up.

Adult patients aged above 18 years having coronary artery disease who underwent bifurcation stenting and were willing to give informed consent were included in the study. Patients with contraindications to antiplatelet therapy, those who had previous stenting of the same bifurcation lesion, those whose CBL had been treated with drug-eluting balloons (DEB) and those unwilling for any intervention or refused to give consent were excluded.

Sample size

Assuming a prevalence of coronary artery bifurcation lesions of approximately 20% [1], the estimated prevalence (p) was taken as 20%, with a corresponding q value of 80%. A 95% confidence interval (CI) was considered, with an allowable error (d) of 10%. The sample size was calculated using the LaTex formula for estimating a proportion:

$n=\frac{Z^2 \cdot p(1-p)}{e^2}$

Where n = required sample size; Z = critical value of the normal distribution (1.96 for 95% confidence level); p = estimated prevalence rate; e = desired margin of error.

After substituting the values, sample size calculated was 61.47 which was rounded off to 70.

A total of 70 patients were enrolled in the study based on predefined eligibility criteria. These patients had angiographically confirmed coronary artery bifurcation lesions and were treated using established bifurcation stenting strategies such as PS, T-stenting, Culotte, Double-Kissing (DK) crush and DK Culotte. The choice of stenting technique was at the discretion of the operators. The procedural success was defined as successful stent deployment with <30% residual stenosis and without in-hospital MACE. All patients received dual antiplatelet therapy for one year and single antiplatelet therapy thereafter. All patients were selected consecutively over the study period, and their clinical characteristics, procedural details, and follow-up outcomes were systematically documented and analysed. They were monitored over one year, with routine follow-up visits scheduled at one-month, six-month and one-year intervals. Additionally, patients were encouraged to report to the cardiology unit at any time if they experienced new or worsening symptoms suggestive of cardiac complications. At each follow-up, medicine compliance was assessed, clinical examination, ECG and echocardiography were repeated to assess cardiac function and detect any adverse events. If clinically warranted, further diagnostic investigations such as stress testing or invasive coronary angiography were performed.

Data analysis

Data were collected and analysed using Statistical Package for Social Sciences (SPSS) version 29. Continuous variables were expressed as mean ± SD and categorical variables, including comorbidities and clinical outcomes, were summarised as frequencies and percentages.

To examine associations between MACE and various comorbid factors following bifurcation stenting, chi-square tests were applied as part of bivariate analysis. Statistical significance was defined as a p-value less than 0.05 for all comparisons performed.

Ethical issues

The study was conducted as par with the ethical principles laid down in the Declaration of Helsinki and was approved by the institutional ethics committee (E/MD/173/1/MS/PT-II/DNB).

## Results

Patient characteristics

A total of 70 patients undergoing PCI for CBLs were analysed. Mean age of the cohort was 65.6 ± 2.8 years, and 72.8% were male (Table 1). In this study population, chronic stable angina (CSA) was the predominant clinical presentation (84.3%). A total of seven patients (10%) had a prior coronary artery bypass graft (CABG), and the mean left ventricular ejection fraction (LVEF) was 45.6%.

**Table 1:** Baseline clinical characteristics of patients undergoing PCI according to lesion characteristics CSA: Chronic stable angina; AWMI: Anterior wall myocardial infarction; IWMI: Inferior wall myocardial infarction; NSTE ACS: Non-ST elevation acute coronary syndrome; MI: Myocardial infarction; CABG: Coronary artery bypass graft; PCI: Percutaneous coronary intervention; LVEF: Left ventricular ejection fraction

Demographic variables	Values
Age (years, mean ± SD; range)	65.6 ± 2.8; 45–78
Gender	N (%)
Male	51 (72.8)
Female	19 (27.1)
Medical history and risk factors	N (%)
Hypertension	50 (71.4)
Diabetes mellitus	45 (64.3)
Dyslipidaemia	28 (40)
Smoking habits	29 (41.4)
Drinks alcohol	24 (34.3)
Obesity	22 (31.4)
Diagnosis	N (%)
CSA	59 (84.3)
AWMI	7 (10)
IWMI	2 (2.8)
NSTE ACS	2 (2.8)
Prior MI	9 (12.8)
Prior CABG	7 (10)
Prior PCI(s)	11 (15.7)
History of congestive heart failure	6 (8.6)
Peripheral vascular disease	5 (7.1)
Cerebrovascular accident	11 (15.7)
Thrombolytics	1 (1.4)
LVEF (%)	45.6 ± 12.9

Angiographic characteristics

The angiographic profile of the study population is summarised in Table 2. The most prevalent CBL subtypes were Medina 1,1,1 (observed in 26 patients (37.1%)) and Medina 1,1,0 (observed in 20 patients (28.6%)), whereas the least common subtype was Medina 0,0,1 (encountered in one patient (1.4%)) (Figure 1). Calcification was seen in 12 cases (17.1%), and thrombus was present in 5 cases (7.1%). The most commonly involved bifurcation sites were the left anterior descending (LAD)/diagonal (n = 24, 34.3%) and left circumflex (LCX)/obtuse marginal (OM) (n = 15, 21.5%). Left main coronary artery (LMCA) to LAD stenting was performed in 8 patients (11.4%), of whom 4 had previously undergone CABG with patent saphenous venous grafts to the major obtuse marginal artery. Similarly, LMCA to LCX stenting was carried out in 5 patients (7.1%), including 2 with patent left internal mammary artery grafts to the LAD.

**Table 2:** Angiographic characteristics of lesions and technique of bifurcation coronary stenting IHD: Ischaemic heart disease; SVD: Single vessel disease; DVD: Double vessel disease; TVD: Triple vessel disease;  MV: Main vessel; LAD: Left anterior descending; LCX: Left circumflex; OM: Obtuse marginal; LMCA: Left main coronary artery; RCA: Right coronary artery; PDA: Posterior descending artery; PLV: Posterolateral vessel; PTCA: Percutaneous transluminal coronary angioplasty; DK: Double-Kissing; TAP: T and small protrusion; SB: Side branch; OCT: Optical coherent tomography; IVUS: Intravascular ultrasound

Variables	N (Percent)
IHD	
SVD	29 (41.4)
DVD	24 (34.3)
TVD	17 (24.3)
MV lesion length	
< 10 mm	23 (32.85)
– 20 mm	20 (28.6)
> 20 mm	27 (38.6)
Vessels treated	
LAD, Diagonal	24 (34.3)
LCX, OM	15 (21.5)
LMCA, LAD	8 (11.4)
Protected LCX	4 (4.7)
RCA, PDA, PLV	8 (11.4)
LMCA, LCX	5 (7.1)
Protected LAD	2 (2.8)
Multivessel PTCA (≥ 2 vessels)	10 (14.3)
LAD-Diagonal and LCX-OM	4 (5.7)
LMCA-LAD and LCX-OM	1 (1.4)
LAD-Diagonal and RCA-PDA	3 (4.3)
LMCA-LCX and LCX-OM	2 (2.8)
Medina classification	
1,1,1	26 (37.1)
1,1,0	20 (28.6)
0,1,1	8 (11.4)
1,0,1	7 (10)
1,0,0	5 (7.1)
0,1,0	3 (4.3)
0,0,1	1 (1.4)
Thrombus	5 (7.1)
Calcium	12 (17.1)
Technique of bifurcation coronary stenting	
Provisional bifurcation	46 (65.7)
DK crush	12 (17.1)
TAP technique	6 (8.6)
Mini crush	4 (5.7)
DK Culotte	2 (2.8)
Mean size of stent used (mm) (mean ± SD )	
MV	3.05 ± 0.32
SB	2.77 ± 0.28
Mean length of the stent used (mm) (mean ± SD )	
MV	23.4 ± 9.9
SB	18 ± 5.6
Intravascular imaging	27 (38.6)
OCT	19 (27.1)
IVUS	8 (11.4)

Coronary bifurcation stenting (COBIS) technique

The most common coronary bifurcation technique utilised was PS (n=46, 65.7%), followed by DK crush technique (n=12, 17.1%), while DK Cullotte technique was least utilised (n=2, 2.8%). Mean size and length of the stents used in main vessel (MV) and SB were 3.05±0.32 mm, 23.4±9.9 mm and 2.77±0.28 mm, 18±5.6 mm, respectively.

Use of intravascular imaging

Intravascular coronary imaging was underused in our study. Optical coherent tomography (OCT) was used in 19 (27.1%) procedures while intravascular ultrasound (IVUS) was used in eight procedures (11.4%).

Outcomes

MACE at one month, six months and one year occurred in 4.3% (95% CI: 1.5-11.9), 7.1% (95% CI: 3.1-15.8) and 7.1% (95% CI: 3.1-15.8) patients, respectively (Table 3). Three patients (4.3% (95% CI:1.5-11.9)) had non-ST elevation myocardial infarction (NSTEMI) at one month, and four patients (5.7% (95% CI: 2.2-14.0)) had NSTEMI at six months and one year, requiring repeat target vessel revascularisation (TVR) with percutaneous transluminal coronary angioplasty (PTCA). One non-cardiac death (1.4% (95% CI:3-7.6)) occurred at six months. Statistical analysis using the chi-square test showed no significant differences in MACE at one, six, and 12 months (χ²=0.65, p=0.72).

**Table 3:** MACE at one month, six months and one year MI: Myocardial infarction; TVR: Target vessel revascularisation; PTCA: Percutaneous transluminal coronary angioplasty; CABG: Coronary artery bypass graft; CI: Confidence interval; MACE: Major adverse cardiac events

Variable/Event	1 month (n)	% (95% CI)	6 months (n)	% (95% CI)	1 year (n)	% (95% CI)
Death, MI, or TVR	3	4.3 (1.5–11.9)	5	7.1 (3.1–15.8)	5	7.1 (3.1–15.8)
Death or MI	3	4.3 (1.5–11.9)	5	7.1 (3.1–15.8)	4	5.7 (2.2–14.0)
Death	0	0.0 (0.0–5.2)	1	1.4 (0.3–7.6)	0	0.0 (0.0–5.2)
MI	3	4.3 (1.5–11.9)	4	5.7 (2.2–14.0)	4	5.7 (2.2–14.0)
TVR	3	4.3 (1.5–11.9)	5	7.1 (3.1–15.8)	4	5.7 (2.2–14.0)
PTCA	3	4.3 (1.5–11.9)	8	11.4 (5.9–20.8)	5	7.1 (3.1–15.8)
CABG	0	0.0 (0.0–5.2)	0	0.0 (0.0–5.2)	0	0.0 (0.0–5.2)
PTCA or CABG	3	4.3 (1.5–11.9)	8	11.4 (5.9–20.8)	5	7.1 (3.1–15.8)

## Discussion

CBLs are among the most challenging subsets in PCI, owing to complex anatomy, variable plaque distribution and the need to protect MV and the SB where their size, their angle of bifurcation can vary. The clinical presentation, like acute coronary syndrome (ACS) or MI, presence of thrombus or calcium make it more complex [9,10]. The present study represents the single-centre, real-world experience of COBIS reflecting contemporary practice patterns along with one-year clinical outcomes among 70 patients. In our cohort, the PS strategy was the predominant approach, consistent with current guideline recommendations advocating a stepwise, conservative technique for most bifurcation lesions. This preference is supported by two large RCTs, the Nordic Bifurcation Study (NORDIC I) and the British Bifurcation Coronary Study (BBC ONE) [11,12]. The European Bifurcation Club (EBC) advocates a stepwise, layered PS approach as the preferred technique for managing CBLs [13]. The increasing use of two-stent strategies in anatomically complex or true bifurcation lesions in our study reflects evolving operator experience and lesion-specific tailoring. Evidence suggests that while overall outcomes between one- and two-stent approaches may be similar, selected subsets, particularly true bifurcation lesions with long SB involvement, may benefit from planned two-stent techniques. In addition, recent network meta-analyses have highlighted that the DK crush technique may offer lower rates of MACE, largely driven by reduced TLR [14]. Our findings, where complex lesions were more frequently managed with two-stent strategies, are consistent with this selective approach.

In this study, the most frequently encountered CBL subtypes was Medina 1,1,1 (37.1%) and Medina 1,1,0 (28.6%) which is comparable to Mohamed et al., where they studied the outcomes of CBL PCI according to Medina Classification [15]. The type of CBL is closely linked to procedural complexity as well as short- and long-term clinical outcomes. In the COBIS II registry, which included 2,897 patients, Park et al. demonstrated that individuals with ‘true’ CBL, particularly Medina subtypes 1,1,1 and 0,1,1, experienced significantly higher rates of adverse events compared with other lesion patterns [16].

An important observation from our study is the effect of lesion complexity on outcomes. Involvement of coronary bifurcations, particularly when the left main artery is affected, has been recognised as an independent predictor of adverse clinical outcomes, including increased mortality [17,18]. Although our study was not powered for subgroup mortality analysis, a trend toward higher event rates in complex bifurcation anatomy was observed, emphasizing the need for careful patient and lesion selection.

Procedural optimisation techniques like FKB inflation, POT, and intravascular imaging (IVUS/OCT) were variably utilised in our cohort. Increasing evidence suggests that these adjunctive strategies improve stent apposition, optimise flow dynamics, and potentially reduce restenosis and stent thrombosis. From a mechanistic standpoint, different stenting techniques produce distinct hemodynamic environments, influencing wall shear stress and restenosis risk. This highlights the importance of meticulous technique beyond the choice of stenting strategy alone.

The evolving role of newer-generation DESs, improved imaging modalities, and refined techniques has contributed to better outcomes in bifurcation PCI [19,20]. Emerging computational and imaging-based models may further enable patient-specific procedural planning, potentially improving outcomes in complex bifurcation lesions.

Limitations

There are some limitations the study has. It is a single-centre study with a limited sample size, which restricts the broader applicability of the findings. Moreover, the absence of randomisation increases the probability of selection and operator bias. Furthermore, procedural approaches were not uniform and were determined at the discretion of individual operators. Lastly, the relatively short duration of follow-up limited the evaluation of late events such as restenosis and stent thrombosis.

## Conclusions

Our single-centre, real-world experience demonstrates that PS remains the default strategy for majority of the CBL, with selective use of two-stent techniques in complex anatomy. Clinical outcomes were comparable between strategies, supporting a lesion-specific, individualised approach. Continued advancements in technique, imaging, and device technology are expected to further improve outcomes in this challenging subset of coronary interventions.
